# Implementation of disability policy framework in Namibia: A qualitative study

**DOI:** 10.4102/sajp.v74i1.400

**Published:** 2018-04-09

**Authors:** Tonderai W. Shumba, Indres Moodley

**Affiliations:** 1 School of Nursing and Public Health, University of KwaZulu-Natal, South Africa

## Abstract

**Background:**

This study explores some of the experiences of national programme managers, heads of national organisations of persons with disabilities (OPDs) and persons with disabilities in the implementation of the disability policies and legal framework in Namibia.

**Method:**

In-depth interviews were conducted with multiple key stakeholders mentioned above. Interviews were digitally recorded and transcribed verbatim. The Community Based Rehabilitation (CBR) matrix (health, education, livelihood, social and empowerment) was utilised to guide the development of themes and subthemes.

**Results:**

Twenty-one key informants participated in the study. Participants stated that while Namibia has made significant progress in addressing the medical and social needs of persons with disabilities, further progress can be achieved through better coordination, capacity building, review and updating of policies which allows for the inclusion of personal assistance, access to justice, improving supply chain management for a wide range of assistive devices, mainstreaming HIV prevention and treatment programmes, improved access to sexual reproduction and family planning, improved access to higher education, curricula reviews and effective monitoring and evaluating of the CBR programme.

**Conclusions:**

The study revealed key issues that need to be addressed in reviewing the policy and legal framework so that it is responsive to the current needs of persons with disabilities. Further, the CBR programme needs an evaluation tool to assess its effectiveness and efficiency in meeting the needs of persons with disabilities and also to elicit their experiences and satisfaction.

## Background

Health care provision in Namibia is guided by the Constitution (Republic of Namibia 1991) which provides that: ‘ … every citizen has a right to fair and reasonable access to public facilities and services in accordance with the law’ (Article 95). The responsibility of providing health services is vested in the Ministry of Health and Social Services (MOHSS). At independence in 1990, Namibia inherited a fragmented, urban- and curative-based health system with a limited preventative health care component. Health services in rural areas, in particular in the northern parts of the country, were provided mainly by the Catholic and Lutheran churches. To redress this deficit and imbalance, the primary health care (PHC) approach was adopted to focus on areas of prevention, health promotion, rehabilitation and also curative care (Ministry of Health and Social Services 2014).

Rehabilitation underpinned by PHC led to the development of policies and legislation to guide the delivery of services for persons with disabilities. In a review of the policy and legislative framework (Shumba & Moodley 2017), four policies were identified that are directly related to disability including the National Policy on Disability (Republic of Namibia 1997), National Policy on Orthopaedic Technical Services (Ministry of Health and Social Services 2001), National Policy on Mental Health (Ministry of Health and Social Services 2005) and Sector Policy on Inclusive Education (Republic of Namibia 2013). Further, the *National Disability Council Act* of 2014 (Republic of Namibia 2004) and the international convention, the United Nations Convention on the Rights of Persons with Disabilities (UNCRPD) (United Nations 2006) ratified in 2007 (Shumba & Moodley 2017) also guided the delivery of these services.

While Namibian policies and legal framework are in place, implementation may not be optimal because of lack of awareness and knowledge of policy content, fragmentation in implementation owing to lack of a central mechanism for coordination, complexity in intersectoral coordination, lack of qualified human resources, ambiguous policy strategies, lack of monitoring and evaluation, budgetary constraints, inconsistent definitions or models of disability and failure to address gender differences (Shumba & Moodley 2017). Similarly, South Africa has a comprehensive disability policy framework, but there is a dichotomy in policy formulation and implementation (Duncan, Sherry & Watson 2011). It appears that there is a universal tendency by governments to create disability policies as a symbolic gesture (Alant, Emmett & Samuels 2007). Thus, most of these disability policies miss the mark because they are interpreted by service providers as tools to deal with ‘problems’ of persons with disabilities and thus fail to accord them their equal rights to enjoy full citizenship (Duncan et al. 2011).

This study explores some of the experiences of national programme managers, heads of national organisations of persons with disabilities (OPDs) and persons with disabilities in the implementation of the disability policies and legal framework in Namibia. While Namibia has made moderate progress in establishing policies and legislation (Shumba & Moodley 2017), there is no evidence of a study conducted to assess the awareness, knowledge and progress of implementation from the perspectives of implementers. This study provides some insights into the status of Namibia disability services and potentially could assist policymakers in identifying areas that need review and strengthening.

## Methods

We conducted a retrospective analysis of progress in implementing policies that directly address the needs and rights of persons with disabilities in Namibia. This study utilised a research design that is qualitative, explorative, descriptive and contextual.

### Participants

A review of policy and legal framework in Namibia (Shumba & Moodley 2017) provided an opportunity for purposive maximum variation sampling to identify key participants in this study. The participants were identified through searching the Namibian government websites and individual referral by staff in each ministry during manual searching of policies and acts. An initial sample size of 12 key participants was identified. However, this list was expanded through snowball sampling for other potential key participants until data saturation was achieved. The final list consisted of 21 participants who are the actual implementers and included national programme managers, heads of national OPDs and selected persons with disabilities. Each participant had 10 or more years of work experience and it was anticipated that they could provide both a retrospective and current reflection based on their experiences.

### Data collection

Interviews were conducted from June 2016 to August 2016 in English, the official language in Namibia. These interviews were conducted at the offices of the selected participants. An information sheet and consent forms were sent to all participants prior to the interview. A semi-structured questionnaire was used to collect data. This consisted of Section A with four closed-ended questions and Section B with three open-ended and non-directive questions. The open-ended and non-directive questions focused on participants` awareness and experiences for the implementation of the policies (Appendix 1). Rewording of the questions was done as necessary, and given the semi-structured nature of the interviews, additional probing was used. All interviews were audio-recorded and lasted between 45 and 60 min.

### Analytic framework

#### Theoretical framework

**FIGURE 1:**
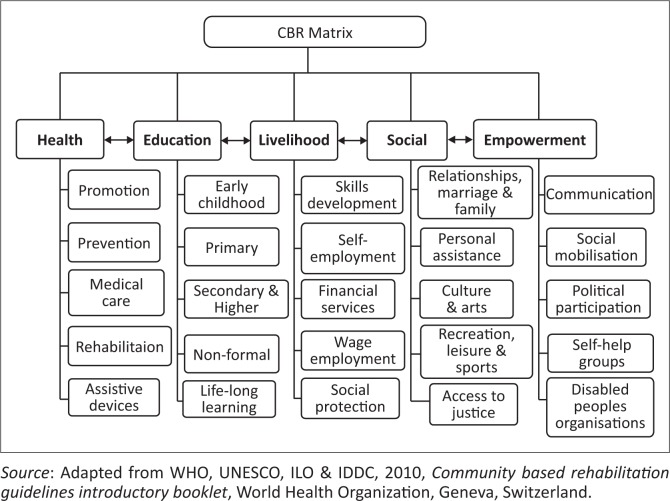
Community Based Rehabilitation matrix.

This study utilised the Community Based Rehabilitation (CBR) matrix (Figure 1) that forms the basis of CBR guidelines (WHO et al. 2010) to guide the analysis. The WHO CBR matrix, which has five key components including health, education, livelihood, social and empowerment formed the basis on which to record the themes and subthemes from the key informant interviews.

The reason for selecting the CBR matrix and CBR guidelines as a theoretical framework for analysis was threefold. Firstly, an initial study on a review of policy and legal framework in Namibia (Shumba & Moodley 2017) identified that CBR underpins the policy and legal framework as the main strategy for the delivery of disability services. Secondly, the CBR guidelines and matrix may enable a ‘society for all’ through targeting sectors of development and ensuring that they are inclusive. Involving all sectors of development has the potential of empowering persons with disabilities with the ultimate aim of improving their quality of life. Thirdly, the CBR guidelines and matrix are resolutely underpinned by the UNCRPD (United Nations 2006) that promotes and enhances the social and human rights model of disability.

#### Transcripts analysis

Audio recordings were transcribed verbatim, replacing any identifying information with pseudonyms. General thematic analysis was undertaken based on the entire set of transcripts. Responses to some closed-ended questions (Question 2, Section B) (Appendix 1) were analysed quantitatively using descriptive statistics.

A preliminary report of the interviews was given to the key informants for review and comments and corrections were made, where applicable. Each theme consisted of statements that best described the detail, thought or idea on a particular topic. To ensure systematic identification of statements for each theme, each transcript was analysed and agreed upon as to which of the statements could be included and those that should be excluded from each particular theme. Disagreements on inclusion and exclusion of statements that best represent a category were resolved through discussion.

Data management and analysis were done using QSR International’s NVivo 11 qualitative analysis software. This involved six steps: data cleaning, uploading the data onto NVivo 11 software, recognising the data, conducting data exploration (using *Query* command), coding relevant information in the data and generating themes.

### Trustworthiness

Trustworthiness was ensured using Lincoln and Guba’s strategies for the qualitative approach including credibility, transferability, dependability and confirmability (Lincoln & Guba 1985). *Credibility* was assured by prolonged engagement until the scope of data was adequately covered and data saturation on progress on policy implementation was obtained; referential adequacy through use of tape recorder and field notes, triangulation through engagement with different stakeholders, member checks on participants’ responses and peer debriefing of themes with the second author were also applied. Purposive sampling and dense descriptions ensured *transferability*. Recorded audiotapes, written field notes, methodological notes and reflexivity ensured *dependability* and *confirmability*.

### Ethical consideration

Ethics approval was obtained from the Human Sciences Ethics Research Committee of the University of KwaZulu-Natal (Reference No: HSS/0646/015D) and approval to conduct research was obtained from the Ministry of Health and Social Services in Namibia (Reference No: 17/3/3).

## Findings

The results are presented according to participants’ demographics, disability programmes, participants’ awareness and knowledge of policy framework, and participants’ experiences regarding implementation of disability programmes.

### Participant demographics

The roles and functions of the 21 participants varied significantly, from representatives of persons with disabilities to a cabinet minister, as shown in Table 1, ensuring a broad spectrum of experiences and responses.

**TABLE 1:** Participants’ characteristics.

Participant ID	Gender	Organisation	Job title	Disability experience
P1	Female	Ministry of Health and Social Services – national level	Acting deputy director – Division Disability Prevention and Rehabilitation	> 15 years
P2	Female	Ministry of Health and Social Services – national level	Former acting CEO, currently senior health programme officer	> 15 years
P3	Male	Ministry of Health and Social Services – Windhoek Central Hospital	Chief prosthesis/orthotics	> 10 years
P4	Female	Ministry of Health and Social Services – national level	Senior health programme officer (National Programme Manager – Mental Health Services)	> 15 years
P5	Female	Ministry of Education – national level	Chief education officer	> 10 years
P6	Male	Ministry of Justice	Chief legal interpreter	> 15 years
P7	Male	Ministry of Poverty Reduction and Social Welfare	Acting deputy director and former National Disability Council member	> 10 years
P8	Male	National Disability Council	Acting deputy director and former National Disability Council member	> 15 years
P9	Male	National Federation of Persons with Disabilities (NFPDN)	Chairperson of Executive Board NFPDN and MOHSS staff	> 15 years
P10	Male	Person with disability	Representative of persons with disability and former secretary general of NFPDN	> 15 years
P11	Male	Volunteer to National Organisations of Persons with Disabilities	Disability consultant and Former Peace Corp volunteer	> 15 years
P12	Female	Office of the Vice President – Disability Affairs	Minister – politician	> 15 years
P13	Female	Ministry of Health and Social Services – Intermediate Hospital Katutura	Occupational therapist	> 10 years
P14	Male	Ministry of Health and Social Services – Intermediate Hospital Katutura	Physiotherapist	> 10 years
P15	Male	National Federation of the Visually Impaired (NFVI)	Human rights and advocacy officer	> 10 years
P16	Male	Namibia National Association of the Deaf (NNAD)	Chairperson of NNAD and school teacher for the deaf	> 10 years
P17	Female	Ministry of Health and Social Services – national level	Chief health programme officer (orthopaedic technical services)	> 15 years
P18	Female	The Association for Children with Language, Speech and Hearing Impairments of Namibia	Director	> 15 years
P19	Female	Ministry of Education – Centre for Communication and Deaf Studies	Chief education officer	> 15 years
P20	Male	Albino Trust of Namibia	Chairperson of Albino Trust and member of National Disability Council	> 15 years
P21	Female	Ministry of Justice and Organisation of National Association of Children with Disabilities (NACD)	Language interpreter (Ministry of Justice), chairperson of NACD	> 15 years

MOHSS, Ministry of Health and Social Services.

### Disability programmes in Namibia

The disability programmes implemented by various agencies are shown in Table 2. Most participants confirmed implementing CBR services for all disability groups or for the specific disability they support (Shumba & Moodley 2017).

**TABLE 2:** Disability programmes and services in Namibia.

Organisation	Disability programmes in place
Ministry of Health and Social Services	Community Based Rehabilitation Institution-based rehabilitation (physiotherapy, occupational therapy, medical rehabilitation and audiology) Visual impairment rehabilitation Mental health Blindness and deafness prevention Orthopaedic technical services
Ministry of Education – national level	Special education (visual impairment, hearing impairment, and intellectual and learning disability) Inclusive education (hearing and visual impairment)
Ministry of Education – Centre for Communication and Deaf Studies	Namibia sign language training Strengthening educational institutions of the deaf Advisory and consulting services of the deaf Research and development of the issues of the deaf Provision of sign language interpreters
Ministry of Justice	No disability-specific programme but are responsible for drafting and interpreting policies and legislation including for disability
Ministry of Poverty Eradication and Social Welfare	Disability grant disbursement under the *National Pension Act*
National Disability Council	Disability awareness in communities Implementation of national policy on disability Entrepreneurship training programme (Memorandum of understanding [MOU] with National University of Science and Technology)
National Federation of Persons with Disabilities in Namibia (NFPDN)	Disability awareness in communities Advocacy for human rights of persons with disabilities
National Federation of the Visually Impaired (NFVI)	Community Based Rehabilitation Mobility training and orientation Computer training Entrepreneurship training Activities of daily living Braille reading and writing Basic English reading and writing Counselling
Namibia National Association of the Deaf	Deaf awareness (HIV and AIDS, disability grants) Sign language training Development of sign language documents (dictionaries) Human rights advocacy (education, Namibian sign language as an indigenous language of the deaf)
The Association for Children with Language, Speech and Hearing Impairments of Namibia	Screening of hearing Provision of hearing aids Language stimulation Parent counselling
Office of the Vice President – Disability Affairs	Database of persons with disabilities Mainstreaming of persons with disabilities Economic upliftment of persons with disabilities, support to organisations of persons with disabilities (OPDs) Disability empowerment programmes Disability resource centres
Albino Trust	Advocacy on disability grants Disability awareness Access to education

### Awareness of disability policies and legislation in place

Participants’ awareness of the existence of different disability policies and legislation was assessed by asking participants to name any of the policies and legislation that directly relates to disability. Overall, there is a general awareness of all the policies in place with some participants failing to identify some critical policies or legislation. The four most known documents are: National Policy on Disability, UNCRPD, *National Disability Council Act* and the Sector Policy on Inclusive Education. The extent of awareness of each document is shown in Table 3.

**TABLE 3:** Awareness on existence of disability-related policy and legislation in Namibia.

Policy/legislation	Number of participants who were aware	Percentage (%) of awareness
National Policy on Disability	21	100
United Nations Convention on the Rights of Persons with Disabilities	17	81
*National Disability Council Act*, 2004 (Act No. 26 of 2004)	15	71
Sector Policy on Inclusive Education	14	67
National Policy for Mental Health	8	38
National Policy on Orthopaedic Technical Services	7	33

All participants (100%) were aware of the National Policy on Disability, with over three-quarters (81%) being aware of the UNCRPD. Despite the National Policy on Mental Health (38%) and the National Orthopaedic Technical Services Policy (33%) being in place for over 15 years, only those participants actually involved in their implementation were aware of their existence. In contrast, despite the Sector Policy on Inclusive Education being in place for just over 2 years, two-thirds of the participants (67%) reported being aware of its existence.

### Participants’ experiences regarding implementation progress

Participants reported on their experiences regarding the progress of implementation of the disability policies according to the main themes and subthemes predetermined by the WHO CBR matrix (WHO et al. 2010) as outlined below.

#### Key theme 1: Health

Progress of implementation for the inclusion of persons with disabilities in health services was reported according to the following subthemes: access to rehabilitation services, health promotion and prevention, assistive devices and medical care.

**Subtheme 1: Access to rehabilitation services:** The interviewees reported that there appears to be a critically low budget allocation for rehabilitation services. Further delivery of rehabilitation services has been hampered by a critical shortage of rehabilitation staff as most are attracted by the private sector. Despite overall shortages of rehabilitation personnel, the orthopaedic technical services have attempted to address the skills gap through sending their staff for qualifying courses and postgraduate courses in other African countries. The categories/codes for access to rehabilitation services and the accompanying participants` responses are presented in Table 4.

**TABLE 4:** Key theme 1: Health services.

Subtheme	Subcategory/code	Participant(s) responses
Access to rehabilitation services	Coordination	‘The national level is not capacitated to coordinate physiotherapy, occupational therapy and medical rehabilitation services. There is only one person at national level at the moment to manage all these services.’ (P14) ‘Our linkage with other sectors is very poor … don’t have good coordination with especially private sector with regard to purchasing of assistive devices’. (P3)
	Finance	‘National Disability Council has not been functional for years due to very low budget allocation … and not able to consult widely with persons with disabilities.’ (P8) ‘Orthopaedic Technical Services are under Directorates with what they call “priority programmes” like HIV/AIDS, TB, Malaria. We receive second priority after these programmes are funded.’ (P17) ‘ … for years mental health services have had no budget at regional and district level. We only have a very small budget at national level.’ (P4)
	Staff shortage	‘Most of the occupational therapists in this country are foreigners mostly from southern Africa who work on contracts. Namibia does not have courses for training rehabilitation professionals.’ (P13) ‘We do not have enough mental health staff at regional and national level … one person at national level is coordinating mental services with no focal person in the regions.’ (P4) ‘ … there are only three audiologists in public sector of whom one is in the MOHSS and the other two in Ministry of Education … in contrast in the private sector where there is about 10 audiologists and two hearing aid acousticians….’ (P18)
	Lack of skills	‘The orthopaedic technical services send staff for training from certificate to Masters level in different categories. Most of our posts are now filled and we are wondering what we will do with the ones that are under training now.’ (P3)
Access to assistive devices	Finance	‘ … we just provide basic assistive devices to our clients. Budgets for orthopaedic technical services have been going down since 2012 from N$20 million, to only N$1 million in 2017.’ (P3) ‘Ministry of Education has no budget for hearing aids for learners and MOHSS has a very small budget only for adult patients, not children.’ (P18) ‘ … bursaries for disabled students do not include a portion for assistive devices. You are given the same N$20 000 for tuition as for able bodied students. A visually impaired student will have to find extra money to buy software for your computer and other assistive devices ….’ (P9)
	Supply chain	‘The tender process says that companies should be 100% Namibian owned. There are no manufacturers of orthopaedic devices in Namibia. We have middle men who put low prices during tender bidding and when asked to supply they fail.’ (P17) ‘We don’t have assistive devices for visually impaired in Namibia. We import and it’s very expensive and difficult to order as an individual. They ask you VAT and other taxes. So we end up using registered welfare organisation that are exempted from these charges at the border.’ (P15) ‘ … wheelchairs and clutches are on government tender but not white canes and hearing aids.’ (P9)
	CBR programme	‘CBR is driven by volunteers who assist in identifying and referring persons in need of assistive devices … this has been helping in remote areas.’ (P1) ‘ … If CBR was in most rural areas, persons with disabilities will gain much … the only challenge I see with is that we have not done any evaluation to see how is it benefitting persons with disabilities … we only collect demographic data and not asking persons with disabilities how they are perceiving this programme.’ (P11)
Access to medical care	Mental health	‘We only have two centres in the country providing mental health treatments, Oshakati Hospital and Windhoek Central Hospital … all patients are sent to these centres which are always full and not having space.’ (P4)
	Lack of Braille and sign language	‘We cannot read prescriptions. We don’t know whether this is Panadol or paracetamol because the prescriptions are not in Braille. We don’t have privacy to our medication.’ (P9) ‘…it’s very difficult for the deaf to access health care due to lack of sign language interpreters and non-deaf personnel who are trained in offering counselling and other important services … information of PHC is missing leading to most deaf would be mothers not getting information on their unborn babies and other issues of concern.’ (P16)
	Signage for persons with visual impairment	‘Namibian health institutions don’t have directions for visually impaired to follow. You can come to the hospital or clinic and unless you are used to move around there, you need assistance to be taken to different services.’ (P9)
	Medical fees	‘We have a circular within the MOHSS which exempts persons with disabilities … the challenge is in some areas the administrators choose not to follow it and make them pay.’ (P2)
Health promotion and prevention	Awareness campaigns	‘…we have spearheaded the National Disability Day and International Day on Persons with Disabilities in collaboration with other line ministries … the Council has a budget for awareness raising every year … health promotion is part of the activities.’ (P8)
	HIV/AIDS and sexual reproduction education	‘Our policies don’t address issues of HIV/AIDs and disabilities.’ (P19) ‘ … National Policy on Disability should have tackled sexual reproduction. Our girls with disabilities are being sexually abused and are not consulted on matters relating to their sexual needs. There are reported cases that I know of mothers who remove the uterus of these girls or inject them to stop them from having babies.’ (P21) ‘Family planning education is crucial to persons with disabilities especially those with mental disabilities, yet it was missed in the National Policy on Disability.’ (P21)

CBR, community based rehabilitation.

**Subtheme 2: Access to assistive devices:** Most participants reported the lack of adequate budget allocation for the provision of assistive devices. Further challenges were revealed including poor supply chain, lack of some assistive devices on government tender, as well as exorbitant import tax when importing assistive devices that are not locally available. Notwithstanding the challenges in accessing assistive devices, CBR programmes were noted as the main vehicle assisting with access to assistive devices in remote areas. The categories/codes for access to assistive devices and the accompanying participants’ responses are presented in Table 4.

**Subtheme 3: Access to medical care:** The study revealed that there is an exemption for persons with disabilities from medical fees. However, this exemption option is not being applied consistently by health workers. Another challenge revealed was the lack of mental health services, with only two mental health centres countrywide. However, the government is making efforts to improve mental health services through the establishment of postgraduate mental health courses at a local university, sending students outside the country for mental health training, and provision of short courses to intern social workers.

Access to medical care for persons with visual and hearing impairments was reported to be hindered by lack of Braille on prescriptions and sign language during doctors’ consultations, respectively. Persons with visual impairments further reported poor signage in most hospitals which allow for ease of physical accessibility. The categories/codes for access to medical care and the accompanying participants’ responses are presented in Table 4.

**Subtheme 4: Health promotion and prevention:** Success has been recorded in this regard with the National Disability Council allocating funds for raising awareness. One key issue that participants pointed out was that the policies and legal framework failed to address HIV and sexual reproductive issues. Most HIV and AIDS and sexual reproduction programmes are not adapted to meet their individual needs. The categories/codes for health promotion and prevention and the accompanying participants’ responses are presented in Table 4.

#### Key theme 2: Education

The key subthemes identified regarding progress in accessing education are early childhood development (ECD), primary and secondary education and higher education.

**Subtheme 1: Early childhood development:** Most participants expressed that a large proportion of children and young people with disabilities are not receiving a comprehensive education. Factors hindering implementation of the policy framework in this regard include few facilities for ECD and discrimination with regard to the refusal by some schools in enrolling children with disabilities. Despite these challenges, there are factors that have enabled implementation of ECD including political will, occupational therapy assessments and support from non-governmental organisations and the private sector. The categories/codes for ECD and the accompanying participants’ responses are presented in Table 5.

**TABLE 5:** Key theme 2: Education.

Subtheme	Subcategory/code	Participant(s) responses
Early childhood development (ECD)	Few facilities for ECD	‘CLASH manages the only kindergarten for children with hearing impairments in this country, only enrolling a maximum of 10 children per year … The government should open more of these kindergartens countrywide with the high demand.’ (P18)
	Stigmatisation	‘We have challenges in preschools whereby parents of the so called “normal children” want to remove their child if they see that the preschool has enrolled a child with disability.’ (P21)
	Success of ECD (political will, occupational therapy assessments, support from non-governmental organisations and the private sector)	‘ … now the situation of ECD is much better. Most of the preschools have been taken over by government and the government is advocating for free education.’ (P5) ‘ … the government has created a post of deaf-teacher assistant to help with learning facilitation in class but this is only in resource schools.’ (P19) ‘ … occupational therapists help with school assessments for children with learning disabilities. We also assist with school placements for all types of disabilities.’ (P13) ‘ … for the deaf children we [*CLASH*] have at our kindergarten, we assist them with school placements when they graduate into primary education.’ (P18)
Primary and secondary education	Medium of communication	‘Although the school setting acknowledges Namibia sign language as a medium of teaching in schools, it is still not recognized as the FIRST language of the Deaf.’ (P16) ‘ … NAMCOL [*Namibia College of Open Learning*] assists grade 10 failures, but it doesn’t have sign language interpreters ….’ (P19)
	Premature launch of sector policy on inclusive education	‘ … no educational support materials for the inclusive education. I feel we prematurely launched the policy without looking closely at the budget and resources needed.’ (P9) ‘ … while the inclusive policy on education advocates for mainstreaming, it’s not happening. The schools are not ready with adequate facilities, qualified teachers and resources to accommodate children with disabilities.’ (P20)
	Collaboration	‘ … our services [*orthopaedic technical services*] should be assisting the Ministry of Education in implementing inclusive policy, but we have poor communication. We design a lot of upper limb prosthesis that can change the life of a child in school, especially with writing.’ (P3)
	Success of sector policy on inclusive education (free education, sign language training, appointment of class assistants for the deaf and advocacy by organisations of persons with disabilities [OPDs])	‘ … the government has done well with providing free education for primary and secondary education … class assistants have been appointed to assist deaf students.’ (P12) ‘ … the University of Namibia [*Khomasdal campus*] is now offering sign language training to postgraduate teachers ….’ (P5) ‘We [*Organisation of Parents with Children with Disabilities*] have lobbied with several schools in Khomas for physical accessibility. At Van Rhyn Primary School, we lobbied for funding to renovate the toilet, at Heriman School, we lobbied for ramps. At Elim School, we pushed for two learners with special needs to be in mainstream classes.’ (P21)
Higher education and vocational training	Limited vocational training courses	‘ … this education system supports boys mostly. Most of the trades at vocational training centres are for boys with disabilities. You only find hair dressing and sewing for girls.’ (P21)
	Reasonable accommodation (sign language, Braille, large print and physical accessibility)	‘I [*person with visual impairment*] have never heard of any private or government college that accommodates persons with hearing and visual impairment. Most institutions are not equipped or just don’t know how to start to enroll us.’ (P9) ‘ … Universities here cannot adequately accommodate persons with visual and hearing [*sic*] impairments except University of Namibia.’ (P5)
	Assessments of examinations	‘ … most persons with disabilities spend lot of years up to 6 years in vocational training. They just give a reason that we don’t know how to assess them with examinations … some end up dropping without being examined.’ (P21) ‘ … persons with albinism suffer when it comes to assignments, books and examinations. All these are not given in large print … spend more time straining eyes in examinations….’ (P20)

**Subtheme 2: Primary and secondary education:** Participants reported that a number of challenges have been encountered regarding primary and secondary education including the medium of communication, poor collaboration, lack of equity in inclusive education and lack of adequate preparation before launching of the Sector Policy on Inclusive Education (SPIE). Notwithstanding these challenges, efforts have been made to enable implementation of the SPIE through the provision of free education, sign language training, the appointment of class assistants for the deaf and advocacy by OPDs. The categories/codes for primary and secondary education and the accompanying participants’ responses are presented in Table 5.

**Subtheme 3: Higher education and vocational training:** Participants identified many challenges in the delivery of higher and vocational education including reasonable accommodation (sign language, Braille, large print and physical accessibility), limited courses for vocational training and lack of guidelines for assessing examinations for persons with different types of disabilities. The categories/codes for higher education and vocational training and the accompanying participants’ responses are presented in Table 5.

#### Key theme 3: Livelihood

The key subthemes elicited under this theme are social protection, employment and skills development.

**Subtheme 1: Social protection:** A few participants noted that the government has instituted a secure social protection system in the form of disability grants. However, disbursement of the disability grant is faced with challenges of inconsistent assessment of disability by state doctors and in some cases abuse of the disability grant by caregivers. The categories/codes for social protection and the accompanying participants’ responses are presented in Table 6.

**TABLE 6:** Key theme 3: Livelihood.

Subtheme	Subcategory/code	Participant(s) responses
Social protection	Success of disability grant	‘ … I must say the political will is there to provide disability grants. … Recently the government even increased the amount of disability grant from N$500 to N$1100. Namibia is amongst very few African countries that provide disability grants.’ (P12) ‘ … we have a central system that has been working well in paying out disability grants.’ (P7)
	Inconsistency in disability grant assessments	‘Doctors support applications for disability grants of some persons but not others. For example, with Albinism, you can go to this doctor they refuse and when you go to the other you can get support….’ (P20)
	Abuse of disability grants	‘ … some children don’t even know they are receiving a disability grant. Government should put a monitoring system to see that the needs of the child are met once the grant is paid out every month.’ (P21)
Employment (wage and self-employment)	Lack of education	‘ … persons with disabilities who have not benefitted from formal education struggle to compete in mainstream employment.’ (P11)
	Lack of reasonable accommodation	‘Reasonable accommodation is important … I know of a friend with visual impairment who graduated with a teaching degree and is still unemployed. She was asked by the Ministry of Education to bring her own assistant then share the salary.’ (P15)
Skills development	Loss of mandate owing to changing ministries	‘Our national policy advocates for sheltered employment and there was one called Ehafo in Windhoek. However, Ehafo which was under Ministry of Lands moved to the Ministry of Education, everything stopped … it lost its mandate.’ (P2)
	Lack of reasonable accommodation	‘ … most of our VTCs are not accessible for the deaf and the hearing impaired.’ (P5)
	Success of skills development	‘ … The National Disability Council has an entrepreneurship programme through an MOU with National University of Science and Technology which started in 2014 … they have trained 28 persons in business, computer science, marketing, English and accounting.’ (P8) ‘ … the National Federation of the Visually Impaired provides training in entrepreneurship. To overcome challenges in getting formal jobs, we give them basic skills to start their own small businesses….’ (P15)

VTC, vocational training centres.

**Subtheme 2: Employment (wage and self-employment):** The key issues identified as limiting employment opportunities for persons with disabilities include lack of education, reasonable accommodation (sign language, Braille, large print, physical accessibility and appropriate assistive devices) and stigmatisation. The categories/codes for employment and the accompanying participants’ responses are presented in Table 6.

**Subtheme 3: Skills development:** Participants indicated that skills development is a challenge. Key issues identified were loss of mandate owing to changes in ministries and lack of reasonable accommodation in vocational training centres (VTC). Despite these challenges, there are some positive initiatives in skills development as reported by some participants such as entrepreneurship training. The categories/codes for skills development and the accompanying participants’ responses are presented in Table 6.

#### Key theme 4: Social

This study found that persons with disabilities do not enjoy full participation and inclusion within their families and communities. Key subthemes elicited responses covering personal assistance and access to justice.

**Subtheme 1: Personal assistance:** Personal assistants are essential in enabling some persons with disabilities to achieve their full participation and inclusion in society. Participants indicated that some key issues hindering the implementation of this subtheme include lack of specific policy provision regarding personal assistance particularly for persons with multiple disabilities and budget limitations. The categories/codes for personal assistance and the accompanying participants’ responses are presented in Table 7.

**TABLE 7:** Key theme 4: Social.

Subtheme	Subcategory/code	Participant(s) responses
Personal assistance	Lack of policy provision	‘ … the question is who needs a personal assistant? … This is not clear in the policy.’ (P12)
	Assistance of persons with multiple disabilities	‘ … we also need to look at personal assistance for those with serious disabilities. If we have funds from the National Disability Council, we could take care of their needs more adequately.’ (P8)
Access to justice	Provision of evidence in court	‘ … in courts, most of the evidence is visual … you are told to touch your suspect when they know you cannot see. The legal system should provide other ways to assist visually impaired to provide evidence in court.’ (P6)
	Physical accessibility in courts	‘ … we had a case of a man on wheelchair who was in the gallery … could not see or be seen by the magistrate during the court case. Our court rooms are not accessible to persons on wheelchair.’ (P21)
	Inaccessibility to information	‘Both the *National Disability Policy* and *NDC* Act are not in Braille and neither are on recorded CDs, so this makes them inaccessible to people with visual impairments.’ (P10)
	Success of accessibility to information	‘When local language court interpreters have court cases for the deaf, we usually call a sign language interpreter.’ (P6) ‘The National Federation for the Visually Impaired have Brailled a lot of copies for UNCRPD but nothing for other policies ….’ (P10)

**Subtheme 2: Access to justice:** Most persons with disabilities remain vulnerable owing to lack of knowledge of their rights enshrined in the policy and legal frameworks. In analysing the participants’ views, a number of issues emerged such as lack of specific policy provisions including the provision of evidence in court for persons with visual impairments and lack of physical accessibility to courtrooms. However, the Ministry of Justice and other non-governmental organisations have made efforts to improve information accessibility for persons with hearing and visual impairments. The categories/codes for justice and the accompanying participants’ responses are presented in Table 7.

#### Key theme 5: Empowerment

Empowerment is a cross-cutting theme for all the other four themes. The key subthemes elicited in this study were advocacy and communication, disabled persons’ organisations and political participation.

**Subtheme 1: Advocacy and communication:** Advocacy and communication are critical in ensuring that the rights of persons with disabilities are upheld. Most of the participants acknowledged that a reasonable amount of advocacy has been conducted by OPDs and the government. Some of these advocacy initiatives include the creation of disability desks in some offices/agencies/ministries and training of politicians on the UNCRPD. The categories/codes for advocacy and communication and the accompanying participants’ responses are presented in Table 8.

**TABLE 8:** Key theme 5: Empowerment.

Subtheme	Subcategory/code	Participant(s) responses
Advocacy and communication	Disability desk in offices/ministries/agencies	‘As OPDs, we lobbied vigorously to have an office at the highest office in the country. We first had the Disability Unit in the Office of the Prime Minister and in 2015, our President created a Disability Office in the Presidency. We have also lobbied for disability desks in some ministries.’ (P9)
	Training of politicians on UNCRPD	‘… we (NFVI) have trained parliamentarians, councilors, mayors to understand the UNCRPD and how they can apply it in Namibia. We are focusing only on Article 5, 9, 24 and 27.’ (P15)
Organisations of persons with disabilities (OPDs)	Declining donor support	‘OPDs have been always supported by donors. Now donors are pulling out because Namibia is considered a middle-income country, and we are struggling. The remaining donors ask for high requirements for their grants that most OPDs don’t have.’ (P9)
	Financing of OPDs	‘ … our national policy needs review to accommodate the financial needs of OPDs.’ (P7)
	Lack of skills	‘ … we have few people with essential credentials to lead the charge. Coupled by persons with disabilities not having benefitted from formal education, they bring less to the table in driving disability programmes.’ (P11)
Political participation	Representation in parliament and government	‘ … the president appointed one person with disability as an MP in 2005. The same person is now a deputy minister in the Office of the Vice President responsible for Disability Affairs.’ (P10)
	Participation in national elections	‘We (visually impaired) have been able to vote in the last elections using digital voting.’ (P9)

UNCRPD, United Nations Convention on the Rights of Persons with Disabilities.

**Subtheme 2: Organisations of persons with disabilities:** Notwithstanding the government’s commitment to OPDs through policy, most of the OPDs have been faced with challenges, including donor dependency and lack of funds and leadership skills. The categories/codes for OPDs and the accompanying participants responses are presented in Table 8.

**Subtheme 3: Political participation:** This study revealed progress in facilitating the political participation of persons with disabilities including advocacy, participation in voting in national elections and accessibility to public buildings. The categories/codes for political participation and the accompanying participants’ responses are presented in Table 8.

## Discussion

The study explored the knowledge and experiences regarding the implementation of the disability policies in Namibia from the perspective of key informants. Namibia has a relatively comprehensive disability policy and legal framework that has been progressively developed since 1990 (Shumba & Moodley 2017). This study revealed both strengths and weaknesses that have been recorded regarding health, education, livelihood, social and empowerment opportunities for persons with disabilities.

Shumba and Moodley (2017) in their review of policy and legal framework in Namibia identified that the CBR strategy underpins policy and legal framework in Namibia. This finding was confirmed by many participants in this study reporting utilising the CBR strategy in implementing disability services. Further, participants reported that CBR is a major vehicle for accessing assistive devices in remote areas of Namibia. Community Based Rehabilitation is a practical, multisectoral strategy that meets the basic needs of persons with disabilities ensuring their access to health, education, livelihood and social opportunities (WHO et al. 2010). However, participants reported the lack of a qualitative evaluative framework to elicit experiences and measure the quality of life of persons with disabilities. The World Report on Disability recommended appropriate tools to fill the research gap of simultaneously measuring the experiences of persons with disabilities and their quality of life (WHO & World Bank 2011).

All participants were aware of the National Policy on Disability and few were aware of the National Policy on Mental Health and the National Policy on Orthopaedic Technical Services. Further, it emerged that some participants had a poor understanding of the contents of the policy documents. Lack of awareness and knowledge of the existing policy documents can be attributed to poor dissemination and lack of policy orientation and training which hinders effective implementation. Duncan et al. (2011) argue that effective implementation at every level is dependent on capacitating users to policy content in order to foster commitment.

### Health services

In critically analysing participants’ responses, it appears that there is an overall dissatisfaction amongst persons with disabilities with regard to access to health services. Critical gaps were noted with regard to access to rehabilitation services, health promotion and prevention, assistive devices and medical care. Coordination emerged as a significant challenge hindering access to health services. Studies have indicated that coordination of services ensures cost-efficient and equitable distribution of resources to persons with disabilities (Antonelli, McAllister & Popp 2009; Kendall & Clapton 2006). Thus, it is important to develop appropriate referral policies and procedures for effective communication systems amongst service providers.

Participants reported a shortage of staff and essential skills sets. Thus, participants recommended the benefits of including postgraduate training in mental health as well as on-the-job training of intern social workers on mental health issues. It is thus important to bridge this gap through mainstreaming disability courses in both undergraduate and postgraduate training as well as conducting continuous awareness training. Thompson, Emrich and Moore (2003) in their study reported positive outcomes of changing the nursing curriculum on the attitudes of nursing students regarding disability issues.

Although the CBR programme has been instrumental in accessing assistive devices to persons with disabilities, participants reported challenges with the supply chain. For example, government tenders do not include a wide range of devices such as white canes for persons with visual impairments. Another supply chain problem reported was the exorbitant import duties. This indicates the need to review the public procurement system of Namibia to be inclusive of and sensitive to the needs of persons with disabilities. To ease the challenge of procurement, several studies have recommended that government procurement policies can create incentives for the industry to adopt technical standards for universally designed technology (Engelen 2009; Seelman 2008). In addition, southern African countries can consider passing resolutions of harmonising procurement policies as is the case with the European Parliament and other bodies within the European Union (European Commission 2008).

The lack of accessibility has been revealed as hampering access to health services for persons with disabilities. Most participants particularly those with visual and hearing impairments revealed the lack of physical environment accommodation (signage and ramps) and information accessibility (Braille, large print and sign language). Similar findings were reported in Uganda where health services lacked both physical and information accessibility for persons with disabilities (Action on Disability and Development 2005). Miscommunication can also impair understanding of health information, medical instructions, prescribed medications and medical and surgical interventions, and can lead to poor adherence to treatment recommendations (Hoffman et al. 2005; O’Hearn 2006; Sullivan, Heng & Cameron 2006). In Namibia, this finding is attributed to lack of accessibility regulations. The study revealed that most buildings and information has become accessible as a result of continuous advocacy from persons with disabilities. Thus, there is pressure for Namibia to develop accessibility regulations. However, caution should be noted when developing accessibility regulations as it requires focus on ‘pull’ factors, rather than the ‘push’ factors of regulation (Maskery 2007).

There is a need to enforce the regulations once they are developed. The National Disability Council of Namibia has the mandate to propose, develop and monitor the implementation of disability regulation and policy. This function can be supplemented by setting up an independent audit committee. The Uganda National Association on Physical Disability created a National Accessibility Audit Team following the passing of accessibility standards (Uganda National Action on Physical Disability 2017). In addition to this, the OPDs in Namibia can potentially serve as a source of information for local building officials to review building plans, safeguarding against a lack of knowledge amongst local municipalities, planners and designers (WHO & World Bank 2011). Reference can be made to Canada where a local action group worked with the local authorities in the assessment of accessibility problems and plans of improvement (Ringaert 2001).

Notwithstanding the success regarding awareness raising campaigns on disability issues, there is a lack of policy provisions on health education on HIV and sexual reproduction. The study revealed that a significant number of persons with intellectual disabilities are sexually active, yet health promotion is lacking. Parents often do not talk about sexual matters to their children with disabilities (Wazakili, Mpofu & Devlieger 2006), particularly since it has been found that adolescents with physical disabilities are more sexually active than their non-disabled counterparts (Maart & Jelsma 2010). These insights indicate the need for a future review of disability policies to include issues of HIV and sexual reproduction. Similarly, review of the CBR guidelines can consider extending their emphasis on HIV and sexual reproduction.

### Education

There is a general trend that children with disabilities are more likely not to attend schools than children without disabilities (Filmer 2008) and this is even more prevalent in poor countries (United Nations Educational, Scientific and Cultural Organization 2009). Enrolment is also dictated by disability type, with more children with physical disabilities attending schools than children with intellectual and sensory impairments (WHO & World Bank 2011). The National Statistics Agency (2016) reported that 87% of children with disabilities aged 0–4 years are not attending early childhood development programmes and the proportion of persons with disabilities without any formal education was high in rural areas (82.3%) compared to urban areas (17.7%). To mitigate these challenges, Namibia adopted the Sector Policy on Inclusive Education in 2013. The study has indicated that to enhance the limited success of this policy, there has been a need to provide free education for all primary and secondary schools, incorporation of sign language training in postgraduate training of teacher education, appointment of class assistants for the deaf and advocacy by OPDs. Community Based Rehabilitation programmes can also be used as a vehicle to advance inclusive education.

However, there is always a tendency to fall into a trap of ‘one-size-fits-all’ regarding inclusive education. Research has revealed that deaf students and those with intellectual impairments have a negative experience in mainstream schools (Fuchs & Fuchs 1994). The World Federation of the Deaf argues that for deaf learners to have the best environment in mainstream schools both students and teachers should be using sign language for all communications. However, this is not feasible for most schools, hence the need to have special schools where deaf learners can have sign language as the only medium of communication (WHO & World Bank 2011). Furthermore, this study has indicated that mainstream schools in Namibia usually face challenges with assessment of examinations of children with sensory impairments including visual impairments. In such cases, there is need to identify alternative options for assessments such as oral examinations for non-readers (WHO & World Bank 2011).

### Livelihood

Success and challenges were recorded regarding social grants, employment and skills development. In terms of social grants, the MOHSS has the mandate of doing assessments through a state doctor to determine eligibility (Namibian citizen or permanent resident aged 16–59 years) for a disability grant. The study revealed a strong commitment of the government in the provision of disability grants. Despite the disability grant being a good safety net for persons with disabilities, the amount and the system used to allocate grants need to be amended. The current disability grant is N$1000 and maintenance grant is N$250 (International Labour Office & Oxford Policy Management South Africa 2014). Some participants recommended a policy review so that it allows for an increase in the disability grant as well as allowing for paying for personal assistants to persons with multiple and severe disabilities.

There has been a general increase from 20 509 to 27 312 during the period 2009–2013 in the number of persons with disabilities in need of disability grants (International Labour Office & Oxford Policy Management South Africa 2014). However, this number of those receiving disability grants may be far from being precise as the definition of disability used in the policy framework of Namibia is not uniform (Shumba & Moodley 2017), as it is being applied inconsistently by state doctors. There is a need to move from using the historical medical criteria of assessment to assessments that focus on needs that relate to functioning, as reflected in the International Classification of Functioning, Disability and Health (ICF) (World Health Organization 2001).

Another critical issue affecting the livelihood of persons with disabilities in Namibia is employment. The World Report on Disability revealed lower employment rates for persons with disabilities than that of the overall population, with ratios varying from lows of 30% in South Africa and 38% in Japan to highs of 81% in Switzerland and 92% in Malawi (WHO & World Bank 2011). In Namibia, the Employment Equity Commission Report (Ministry of Labour 2014) reported that of the 167 502 people who were employed across all employment sectors, only 5659 were persons with disabilities. Further, contrary to the National Policy on Disability which calls for women with disabilities to be taken as a special target group and Article 6 of the UNCRPDs that requires countries to ensure that women enjoy full human rights, the Employment Equity Commission Report (Ministry of Labour 2014) shows that only 0.2% of women with disabilities are in employment. These lower rates of employment, as most of the participants expressed, are attributed to lack of reasonable accommodation and low educational levels. When chances of securing employment are limited, it often leads to poverty. These statements are supported by WHO, UNESCO and ILO (2004) when they stated that there is a strong correlation between disability and poverty.

### Social

The mission of the National Policy on Disability is to achieve social integration of persons with disabilities. The study revealed key aspects that can promote social integration including personal assistance and access to justice. Although the National Policy on Disability requires that persons with disabilities be provided with personal assistance, it does not provide a clear definition of what constitutes personal assistance. This has resulted in failure and inconsistencies in provision of personal assistance. Thus, appropriate personal assistance schemes should be developed and be inclusive of all types of disabilities (WHO & World Bank 2011). However, other options can be utilised to strengthen personal assistance including the use of CBR strategy and community, home-based care in strengthening personal assistance at the community level.

Access to justice is an enabler for upholding human rights that can promote social integration. The justice system has made progress regarding access to information including use of braille in court documents and provision of sign language interpreters for the deaf during court sessions. However, the study revealed that provision of accessible evidence for the visually impaired and structural adjustments of courtrooms remains a challenge. Article 2 of the UNCRPD states that disability rights litigation also requires the member states to enforce reasonable accommodation for people with disabilities.

There are no provisions in the Constitution of Namibia that explicitly address disability (Shumba & Moodley 2017). Further, disability is not listed as one of the prohibited grounds of discrimination (Ntinda 2013). This study noted cases of discrimination in employment and in schools, and it remains a grey area as to whether courts entertain cases of discrimination. Thus, there is a need for Namibia to pass anti-discrimination laws as is the case with Australia (1992), Canada (1986, 1995), New Zealand (1993) and the United States (1990) (WHO & World Bank 2011). Alternatively, anti-discrimination clauses can either be included in existing legislation like in Germany and South Africa (Mont 2004) or as clauses in constitutions like that of Brazil and Ghana (Degener 2005).

### Empowerment

The study recognised good progress regarding empowerment. Key successes were reported in advocacy and political participation. Namibia has made progress with the representation of persons with disabilities at national, regional and district level as stipulated in Section 3 of the National Policy on Disability. One of the celebrated achievements is the appointment of a person with a disability as a Member of Parliament and the creation of a Department of Disability Affairs in the Office of the Presidency in 2015.

Although OPDs are playing a vital role in advocacy related to disability issues, the study noted financial constraints as all OPDs are dependent on donor support. Most of the donor support has reduced significantly and sustainability of OPDs is threatened. The OPDs called for more government support to ensure sustainability. This may include inclusion of clauses in existing legislation for funding OPDs.

## Conclusion

This study showed that while Namibia has made significant progress in addressing the medical and social needs of persons with disabilities, further progress can be achieved through taking note of the following key issues: policy training; better coordination; review and updating policies; improving supply chain management; developing personal assistance schemes; development of accessibility standards; inclusion of anti-discrimination clauses; mainstreaming HIV and sexual reproduction programmes; increasing funding for OPDs; standardising disability definitions and consideration of adopting the ICF for disability grant assessments; and inclusion of disability studies in undergraduate and postgraduate training.

Community Based Rehabilitation underpins the delivery of disability and rehabilitation programmes in Namibia. However, those interviewed reported that the current monitoring and evaluation system only measures demographic information, including the number of persons with disabilities, types of disabilities and gender of persons with disabilities. The current monitoring and evaluation process does not allow for eliciting the experiences of persons with disabilities in evaluating the effectiveness of the CBR programme. Thus, future research could potentially focus on developing a comprehensive monitoring and evaluation system which also measures the experiences and quality of life of persons with disabilities.

Major gaps in the implementation of disability policies in Namibia are attributed to limited accountability. It appears that the government has not adequately provided the means to be answerable to persons with disabilities regarding health, education, social and empowerment opportunities. This lack of accountability explains why Namibia has a dichotomy between policy formulation and implementation. Implementation is hampered if the policy does not include accountability for service provision (Gilson & Erasmus 2008).

The starting point to revise the policy and legal framework is to include social and legal accountability. Allan (2008) states that social accountability includes aspects like soliciting inputs from a broad cross-section of stakeholders, evaluation of strategic plans, budget reviews, expenditure tracking reports, service delivery reports, analyses of cases of misuse or abuse of resources and accountability in the form of an oversight report. Legal accountability as stated by Aldersey and Turnbull (2011) ensures that courts uphold the rights of persons with disabilities. Furthermore, Aldersey and Turnbull (2011) propose a mixture of both social and legal accountabilities in future policy adoptions or modifications.

## APPENDIX 1

### Semi-structured questionnaire

Section A

1. Name of organisation:
2. Position of interviewee:
3. Gender:
4. Years of experience in disability field:

Section B

1. Identify the disability programmes/services that you are implementing in your department/organisation/ministry/agency.
2. Indicate the policies/Acts you are aware of that directly relates to disability and that have been helping you to implement the programmes you mentioned.
3. From your experiences, what are your comments/observations on the progress of disability policy implementation in Namibia?
  Guiding areas:
  (a) Health services
    Probe: rehabilitation, assistive devices, medical care, health promotion
  (b) Education services
    Probe: early childhood development, primary education, secondary and higher, tertiary, lifelong education
  (c) Livelihood services
    Probe: social protection, skills development, employment, financial assistance
  (d) Social services
    Probe: personal assistance, justice, marriages, sports and leisure,
  (e) Empowerment services
    Probe: political participation, advocacy and communication, self-help groups, OPDs, social mobilisation
